# Ablative Radiotherapy as a Noninvasive Alternative to Catheter Ablation for Cardiac Arrhythmias

**DOI:** 10.1007/s11886-017-0886-2

**Published:** 2017-07-27

**Authors:** Paul C. Zei, Scott Soltys

**Affiliations:** 1 Department of Medicine, Electrophysiology Section, Brigham and Women’s Hospital, 70 Francis Street, Shapiro Building – Room 05088, Boston, MA 02115 USA; 2 Department of Radiation Oncology, Stanford University, Stanford, CA USA

**Keywords:** Ablation, Stereotactic, Noninvasive, Ventricular tachycardia, Atrial fibrillation, Radiosurgery

## Abstract

**Purpose of Review:**

Stereotactic radioablation is a commonly utilized technology to noninvasively treat solid tumors with precision and efficacy. Using a robotic arm mounted delivery system, multiple low-dose ionizing radiation beams are delivered from multiple angles, concentrating ablative energy at the target tissue. Recently, this technology has been evaluated for treatment of cardiac arrhythmias. This review will present the basic underlying principles, proof-of-principle studies, and clinical experience with stereotactic arrhythmia radioablation.

**Recent Findings:**

Most recently, stereotactic radioablation has been used to safely and effectively treat a limited number of patients with malignant arrhythmias, including ventricular tachycardia (VT) and atrial fibrillation (AF). Treatment protocols, outcomes, ongoing studies, and future directions will be discussed.

**Summary:**

Stereotactic radioablation is a well-established technology that has been shown to be a safe and effective therapy for patients with drug-refractory cardiac arrhythmias, including VT and AF. Further clinical evaluation to define safety and efficacy in larger populations of patients is needed.

## Introduction: Current Therapy for Cardiac Arrhythmias—Limitations and the Need for Better Therapies

The underlying anatomic substrates that initiate and/or maintain cardiac arrhythmias have been largely elucidated [1]. These anatomic structures may be congenitally present, as in the case of an accessory pathway, may be the result of pathologic changes to the heart tissue, as in post-myocardial infarction-related scar in many ventricular tachycardias (VT), or possibly a combination of these mechanisms, as postulated for abnormal pulmonary vein activity in patients with atrial fibrillation (AF). Antiarrhythmic drug therapy targeting these substrates has been available for many years, but inadequate efficacy and side effects have limited their use. Interventional therapies, specifically catheter ablation, that disrupt or alter the underlying anatomic arrhythmogenic substrate have become the mainstay of therapy. Most ablative techniques currently utilize radiofrequency (RF) energy to heat tissue in direct contact or close proximity to the tip of a catheter electrode, leading to tissue necrosis, and if successful, disruption of the underlying arrhythmia substrate. Cryoablation, which utilizes a freezant within the catheter to destroy targeted tissue in direct contact with the catheter, has gained increased utilization recently [1].

Catheter ablation can be performed for most tachyarrhythmias. Success rates and complication risks vary quite significantly between procedure types, highly dependent upon the underlying substrate targeted. Among targets for catheter-based ablation, VT, and to some extent AF, have the lowest reported success rates [2, 3]. Failure in patients undergoing catheter ablation for VT or AF is usually due to a handful of commonly recurring issues. Inadequate heating and subsequent scar formation at the desired ablation target can lead to the return of electrical activity in these tissues, as is postulated for pulmonary vein reconnection after initially successful AF ablation [2, 3]. Oftentimes, the larger the substrate targeted, and in turn the larger the ablation lesion set created, the more likely there will be recurrence due to tissue recovery of electrical conduction. The decision to not deliver ablative energy to a target site due to its close proximity to a critical structure is another common cause for ablation failure; concerning sites include critical conduction system structures such as the sinus or AV nodes, the esophagus or phrenic nerve, and the phrenic nerve or coronary artery vasculature during epicardial VT ablation [2, 3]. Perhaps the most common cause of catheter ablation failure is an arrhythmia substrate location that is inaccessible using catheter-based ablation techniques, typically due to a location deep to the endocardial and/or epicardial surfaces of the myocardium [2].

Given the limited ability to disrupt some of these deep substrates, alternative ablative modalities and techniques have been developed in recent years. Alternative therapeutic options available for these “deep” substrates include ETOH ablation, needle ablation, or bipolar ablation. With ETOH ablation, the coronary artery distribution supplying the target substrate is identified, and ETOH is injected into that coronary branch, resulting in infarction of the target and surrounding myocardium [4]. Though potentially quite effective, the most concerning limitations of this approach are the inability to control the extent of infarcted tissue, or conversely the inability to completely infarct the intended target. A specialized catheter that incorporates an extendable/retractable injection needle at its tip allows for delivery of irrigated RF energy through said needle when impaled into the myocardium [5]. This allows for RF delivery deep within the myocardium, potentially reaching and disrupting deep intramural substrates. Although potentially quite effective and potent, this technique requires precise mapping, and it may have an increased risk of cardiac perforation and tamponade. With so-called bipolar ablation, two ablation catheters placed in close proximity straddling the intended substrate target allow more directed and concentrated RF energy to be delivered to targeted deep substrates [6]. Although reportedly effective in many instances, bipolar ablation is technically challenging and may yet not be able to adequately disrupt deep, large substrates.

## Radiotherapy and Stereotactic Ablative Radiotherapy: General Principles and Current Applications

Unlike catheter-based ablation, which utilizes either RF energy to heat tissue or freezant to injure tissues, radiotherapy utilizes photons from X-rays or gamma rays to affect targeted tissue, most commonly cancer. Through modern delivery techniques such as intensity-modulated radiotherapy (IMRT), a dose of radiotherapy can be accurately and precisely delivered to the intended target, while minimizing dose to adjacent normal anatomic structures. Unlike the single point or planar geometry construct of catheter-based ablation, radiotherapy targets a three-dimensional volume defined by the radiation oncologist. Historically, radiotherapy for definitive treatment of cancer has consisted of multiple daily fractions of irradiation over 4–8 weeks, at a dose of approximately 2 Gy per day, leading to high rates of cure for a variety of histologies, including lymphoma, sarcoma, and cancers of the prostate, lung, head and neck, and cervix. With traditional techniques, fractionation of radiotherapy over several weeks is required, as the targeted tumor is adjacent to normal critical structures; simplistically, normal tissues repair the damage from radiotherapy to a greater extent than tumor, leading to greater injury to the tumor compared to normal organs over time.

A change in the fractionated radiotherapy treatment paradigm occurred with the advent of stereotactic radiosurgery in the brain [7]. Radiosurgery consists of 1–5 fractions of very high-dose radiotherapy (e.g., 16–25 Gy in 1 fraction or 25–40 Gy in 5 fractions for brain tumors), delivered with stereotactic accuracy. Compared to fractionated irradiation, whose intent is incremental tumor cell kill over many weeks, the intent of radiosurgery is ablation of the targeted tissue with a minimal number of treatment sessions. Submillimeter accuracy is required, as these high doses would ablate both normal tissue as well as tumor.

The application of these radiosurgical principles outside of the brain is termed stereotactic body radiotherapy (SBRT) or stereotactic ablative radiotherapy (SABR), used for ablative treatment for tumors in the spine, lung, liver, prostate, pancreas, and other sites. Complex treatment planning and delivery are essential to ablate the target while minimizing injury to surrounding non-targeted tissue. Computer-optimized intensities create three-dimensional dose sculpting, concentrating ablative doses to the target, with a rapid dose falloff to minimize toxicity to surrounding tissues. Therefore, unlike traditional external beam radiotherapy which targets both normal structures and the target, exposure of surrounding non-targeted tissues is minimized. Safe delivery requires a multi-disciplinary team including radiation oncologists, radiation physicists, dosimetrists, therapists, and surgeons as indicated.

Additionally, ablative radiotherapy is used in non-oncologic indications, such as trigeminal neuralgia, arteriovenous malformations, seizures, and psychiatric disorders in the brain, and has been explored for renal artery hypertension, back pain, and cardiac arrhythmias, termed stereotactic arrhythmia radioablation (STAR) [8, 9••].

Ablative radiotherapy is typically a noninvasive, outpatient procedure, which does not require anesthesia; the target can be ablated without instrumentation in the patient’s body. Potential risks include injury to structures adjacent to the ablated target, such as brain edema for intracranial targets, lung pneumonitis or esophagitis for thoracic treatments, myelopathy for spinal tumors, or bowel perforation for abdominal sites.

While fractionated radiotherapy leads to cell death primarily through formation of DNA double-strand breaks leading to a replicative cell death, the mechanism of injury for high doses of radiotherapy is less well known and debated [10]. A possible mechanism of ablative radiotherapy is a combination of vascular injury, leading to tissue hypoxia or necrosis, and apoptotic cell death, resulting in fibrosis and scar formation [10]. However, these postulates relate to radioablative injury in malignant tumor cells. Even less is known about the cellular injury mechanisms that may be specific to otherwise normal cells, and in particular normal or at the very least non-malignant arrhythmogenic cardiac tissues. In contrast to RF or cryoablation, the injury from ablative radiotherapy evolves over days to months, taking time for the full tissue injury to manifest. This may play an important role in determining the applications, workflow, and expectations for radioablative techniques.

Once a radiotherapy plan is approved by the treating physicians, irradiation is delivered by a variety of linear accelerator devices. Each of the commercially available systems uses CT or X-ray imaging to ensure accurate patient positioning prior and during treatment. Patients may move during treatment, requiring re-alignment. Additionally, the internal location of the target due to respiratory, cardiac, or bowel-associated movement must be accounted for in the treatment planning and delivery. Interfraction motion management may be accomplished in a variety of manners, from plans that fully encompass the movement of the target to respiratory gating, where the dose is delivered only when the target is within a defined region of the respiratory cycle, to respiratory tracking, where the target is tracked throughout the cycle. Oftentimes, a radio-opaque fiducial marker serves as a surrogate for the target position and used to image movement with X-ray or CT imaging. Treatment times vary from 10 to 90 min based on the machine utilized, target size, and location. The treatment is painless and requires no invasive therapies or sedation.

## Application of Ablative Radiotherapy for Cardiac Arrhythmias

In addition to the typical solid tumors, intracardiac malignancies have been effectively and safely treated with stereotactic radioablation [11]. These same principles can be applied to cardiac ablation for non-oncologic conditions such as arrhythmia. As described, in addition to the respiratory motion compensation typical for thoracic treatment, target movement due to cardiac contraction adds an additional level of complexity in treatment planning. Cardiac motion itself does not result in significant translational movement, as most of the forceful motion occurs in the ventricles, where contraction occurs in a “wringing” motion [12]. In particular, in severe cardiomyopathy, the underlying condition in most patients undergoing VT ablation, overall cardiac motion is even less due to reduced contractility. The atrial structures, particularly the left atrium, exhibit relatively small amounts of translational movement, as the LA is attached to the retroperitoneum via the pulmonary veins. Therefore, respiratory motion resulting in superior-inferior displacement accounts for the vast majority of target motion for intracardiac targets [12].

To compensate for respiratory motion in STAR, a fiducial marker may be localized near the cardiac target. Potentially any radiologically discrete structure that moves in tandem with the target can be used, including in situ foreign structures, in situ native structures, or temporarily placed markers. For instance, in placing a temporary pacing wire used as a fiducial marker, for AF ablation, an RA septal position is targeted; for LV apical VT, an RV apical placement is targeted, while for a basal LV VT, the coronary sinus is targeted. However, several cases have been performed safely and effectively without temporary fiducial placement [13]. In this circumstance, intracardiac and extracardiac native or implanted structures could also be used as a fiducial landmark. In theory, coronary stents, mechanical valves, indwelling pacing leads, coronary or valvular calcification, the diaphragm, or even vertebral or rib bony borders can be used in this way.

After identification of an appropriate patient, a means of defining the anatomic arrhythmia substrate is required. This can be achieved through scar imaging modalities, including nuclear perfusion imaging, cardiac MRI, detailed transthoracic echocardiography delineating wall motion, CT angiography, and in particular electroanatomic mapping during EP study and ablation. With the exception of the latter, successful substrate targeting using the above imaging modalities also most likely benefits from a physiologic correlate; that is, demonstration of electrophysiological evidence that the clinical pathologic arrhythmia indeed likely originates from the anatomically defined substrate. Twelve-lead ECG is a simple tool that can localize VT substrate, typical atrial flutter, PVC origin, accessory pathway location, or even some atrial tachycardia substrates with good accuracy [14], and this alone may be sufficient. Otherwise, prior intracardiac mapping, noninvasive body surface mapping, or in the end simply relying on knowledge of the likely anatomic substrate may prove sufficient. Once imaging and/or electroanatomic mapping have been performed, a treatment delivery plan is constructed off-line by the radiation oncologist, radiation physicist or dosimetrist, and electrophysiologist based on the structural location information provided by the imaging and the electrophysiologic and anatomic localization provided by mapping or electrocardiographic techniques. This planning is typically performed using software designed specifically for the stereotactic radiotherapy platform being used, and in the case of the planning electrophygiologist, primarily involves “drawing” an intended ablation lesion volume on a three-dimensional rendering of the planned target.

Like most radiotherapy treatments, the patient is placed supine on the treatment table and made comfortable to minimize patient movement for the duration of treatment delivery. If the patient needs to get up off the table for any reason, treatment can be interrupted and resumed after proper re-alignment. Once completed, the patient is typically able to immediately ambulate. If there is a temporary fiducial marker in situ, it can be immediately removed afterwards. Patients can then return home after brief observation.

Ablative radiotherapy plans can be accurately delivered without fiducial guidance with proper planning consisting of 4D-CT imaging, where the position of the thoracic structures is determined throughout the patient’s full respiratory cycle. The irradiation may then be delivered via respiratory gating or a breath-hold technique, using the diaphragm as a surrogate for target position within the respiratory cycle. STAR for the treatment of cardiac arrhythmias poses interesting contrasts to traditional catheter or surgical ablation. In addition to the differences in mechanisms of tissue injury, the key pathophysiologic principles of radioablation, in contrast to catheter ablation, are outlined in Table 1.

**Table 1:** Shown are key differences between conventional catheter ablation and stereotactic arrhythmia radioablation

	Catheter ablation	Stereotactic arrhythmia radioablation
Energy source	Radiofrequency energy, cryoablation, others	Ionizing radiation
Pathophysiology of tissue destruction	Tissue heating or freezing leading to necrosis	Likely apoptosis and micro-vascular injury
Time course for lesion formation	Seconds to minutes	Days to months
Tissues that can be targeted	Local injury to tissue in direct contact or close-by proximity to ablative catheter; therefore, tissues accessible by catheter can be ablated	Any tissue is theoretically ablatable
Lesion geometry	Point lesions or planar lesions in contact with catheters	3-dimensional volume
Benefits	• Established technology • Near-immediate effect	• Noninvasive • Any tissue is potentially ablatable, including deep targets • 3D ablation volume can be created
Risks	• Invasive risks • Collateral injury	• Radiation injury • Unknown late side effects

## Stereotactic Radioablation for Cardiac Arrhythmia: Validation and Proof of Principle

**Fig. 1:**
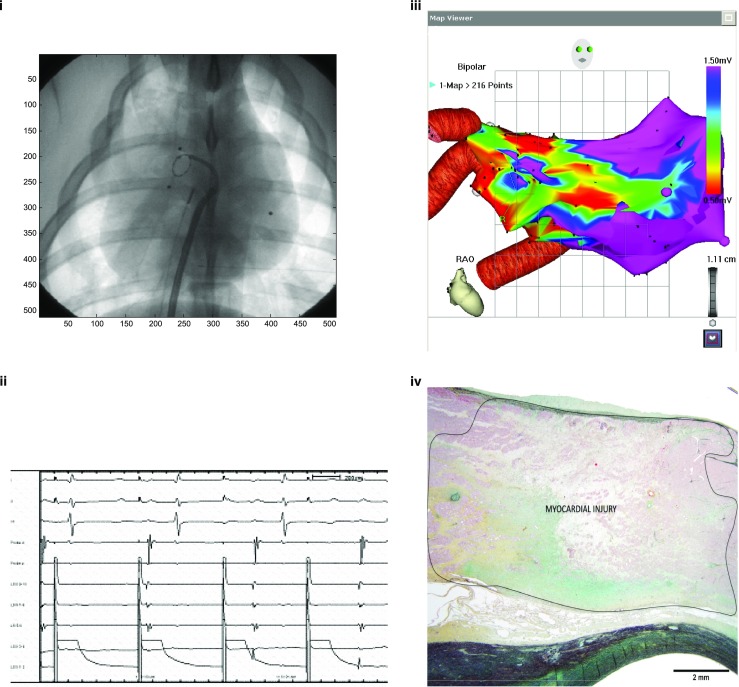
Delivery of 25 Gy single-dose radiotherapy via a stereotactic device in a porcine model. Targeting of the right pulmonary vein antrum results in electrical block of the pulmonary veins, as demonstrated by (*i*–*ii*) exit block seen with high-output pacing in the right pulmonary veins via a circular mapping catheter, (*iii*) electroanatomic mapping evidence of low voltage, and (*iv*) histological staining of anatomic specimens demonstrating evidence of transmural fibrosis at the site of ablation

**Fig. 2:**
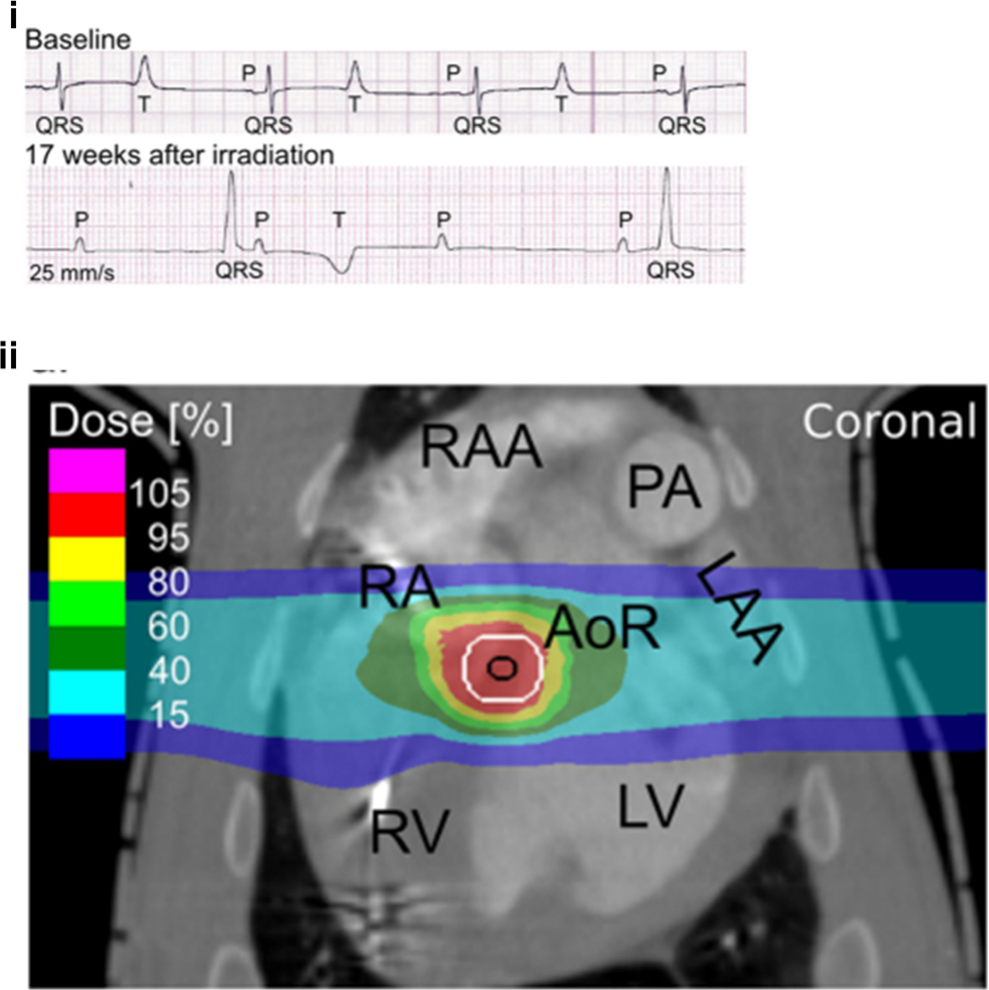
Carbon particle therapy is delivered to the AV node. (*i*) AV block is demonstrated electrocardiographically 17 weeks after carbon particle treatment. (*ii*) Treatment isodose curves shown superimposed on a coronal CT image of the central cardiac structures. Concentrated dosing is shown at the presumed site of the AV node. (Figures are courtesy of H. Immo Lehmann, MD. They are from: Lehmann HI, et al. Sci Rep. 2016 Dec 20;6:38895. doi: 10.1038/srep38895) [17]

In preclinical studies, proof-of-principle was demonstrated by targeting easily defined structures and evaluating easily defined, discrete outcomes. Our group demonstrated successful AV node ablation with STAR, targeted with 25 Gy via a single delivered dose, with AV block demonstrated electrophysiologically [15]. Electrical isolation of pulmonary veins has been demonstrated using pulmonary vein pacing confirming exit block (Fig. 1(i–ii)), electroanatomic mapping (Fig. 1(iii)), and histologic evaluation of ablated tissue demonstrating transmural scar formation after STAR therapy (Fig. 1(iv)) [16]. Similarly, a linear accelerator-based carbon particle beam ablation system has been utilized to successfully target myocardial tissues in an animal model, achieving intended AV block, as shown in Fig. 2, with (i) showing ECG evidence of AV block and (ii) demonstrating the isodose curves for delivered therapy superimposed on a CT scan of the heart encompassing the AV node [18•]. The biophysical characteristics of the interaction between carbon particles and biologic tissues may differ when compared to X-ray-based ionizing radiation; these differences have yet to be elucidated.

In addition to demonstration of efficacy, dose-finding studies have defined ranges of effective therapy delivery and toxicity thresholds (unpublished data). Importantly, no radioablative treatment-related complications were seen both with clinical and pathologic/histologic evaluation in treatments up to 35 Gy. Based on these data, as well as correlates with delivered therapies in the oncology literature and experience, 25 Gy delivered as a single dose has been used in nearly all clinical treatments, even though higher dosing was found to be safe in preclinical studies [15•, 16, 18•, 19]. At this time, the optimal dose in humans remains undefined.

## Clinical Treatment Design and Consideration

The most recent available data as of this publication confirms a total of 3908 and 11,568 radioablation devices installed and in active use in the USA and worldwide, respectively. This includes 3671 USA and 11,015 total worldwide Varian Linac devices, 55 USA and 202 total worldwide Elekta Axesse devices, and 150 USA and 301 total worldwide Accuray CyberKnife devices. In addition, there are 32 USA and 68 worldwide proton beam devices installed [19]. Suffice it to say, there are likely ample facilities to provide cardiac arrhythmia radioablation therapy. The typical treatment protocol first requires a clearly defined patient population. To date, the majority of patients treated have intractable ventricular tachycardia, have failed prior conventional catheter ablation or are not candidates for catheter ablation, and have a defined anatomic arrhythmia substrate amenable to radioablation. Patients have also been treated for drug-refractory AF. In the future, STAR may be a reasonable option for many patients under consideration for ablative therapy for arrhythmias, but currently, this modality has been utilized primarily in patients who have failed other approaches.

In contrast to catheter-based or surgical ablation, STAR treatment effect, specifically scar maturation and reduction and/or elimination of arrhythmia likely occurs over the course of several days to months. Monitoring for arrhythmia burden can be performed as per standard clinical methods. As of current technology, there are no methods to noninvasively evaluate for electrical block across treatment lines, but evidence of scar can be demonstrated with imaging, including CT, PET, MRI, or perhaps noninvasive body surface mapping. Furthermore, noninvasive programmed stimulation protocols are possible in patients with in situ ICDs and pacemakers to test for non-inducibility of arrhythmia. Otherwise, clinical observation of arrhythmia elimination or reduction is the primary endpoint measured. Given the very small number of clinically treated cases so far, the overall long-term success of STAR is unknown.

Procedural risks are typically extremely low due to the noninvasive nature of treatment, and if present, are most often delayed given the nature of tissue injury seen from radiation energy delivery. Theoretical concerns primarily center around misalignment of the intended and actual targets, resulting in excessive dose delivered to surrounding structures, as well as under-dosing of the intended target. Therefore, theoretical complication risks are dependent on the specific cardiac structure(s) targeted. Based primarily on data from mediastinal injury resulting from mantle irradiation for Hodgkin’s Lymphoma therapy, intracardiac injury may include valvular injury, premature coronary disease, pericardial disease, and rarely intramyocardial injury. Phrenic nerve injury, esophageal injury, and pulmonary injury are also possible [20]. As is well-described in the radiation oncology literature, injury risk to other organs not only is dependent on dose of radiation but also tissue-specific vulnerability to radiation injury. However, none of these complications have been demonstrated in both preclinical and clinical evaluations to date using current dosing protocols.

## Clinical Experience

To date, there have been reported eight patients treated for VT and two patients treated for AF with stereotactic radioablation [9••, 13, 16]. Among treated VT patients, five have been treated with the Varian Linac system, while three have been treated with the Accuray CyberKnife system. All VT patients had either failed prior conventional catheter-based VT ablation or were contraindicated for catheter or surgical ablation. Underlying arrhythmia substrates included patients with scar-based VT due to infarct or non-ischemic cardiomyopathy, and idiopathic VT. AF patients all had no prior catheter ablations but had failed medical antiarrhythmic therapy. All patients were treated with 25 Gy delivered in a single dose. Patients all underwent preprocedural imaging to delineate target anatomy. Those who had prior electroanatomic (EA) mapping had their EA maps incorporated into the treatment planning.

**Fig. 3:**
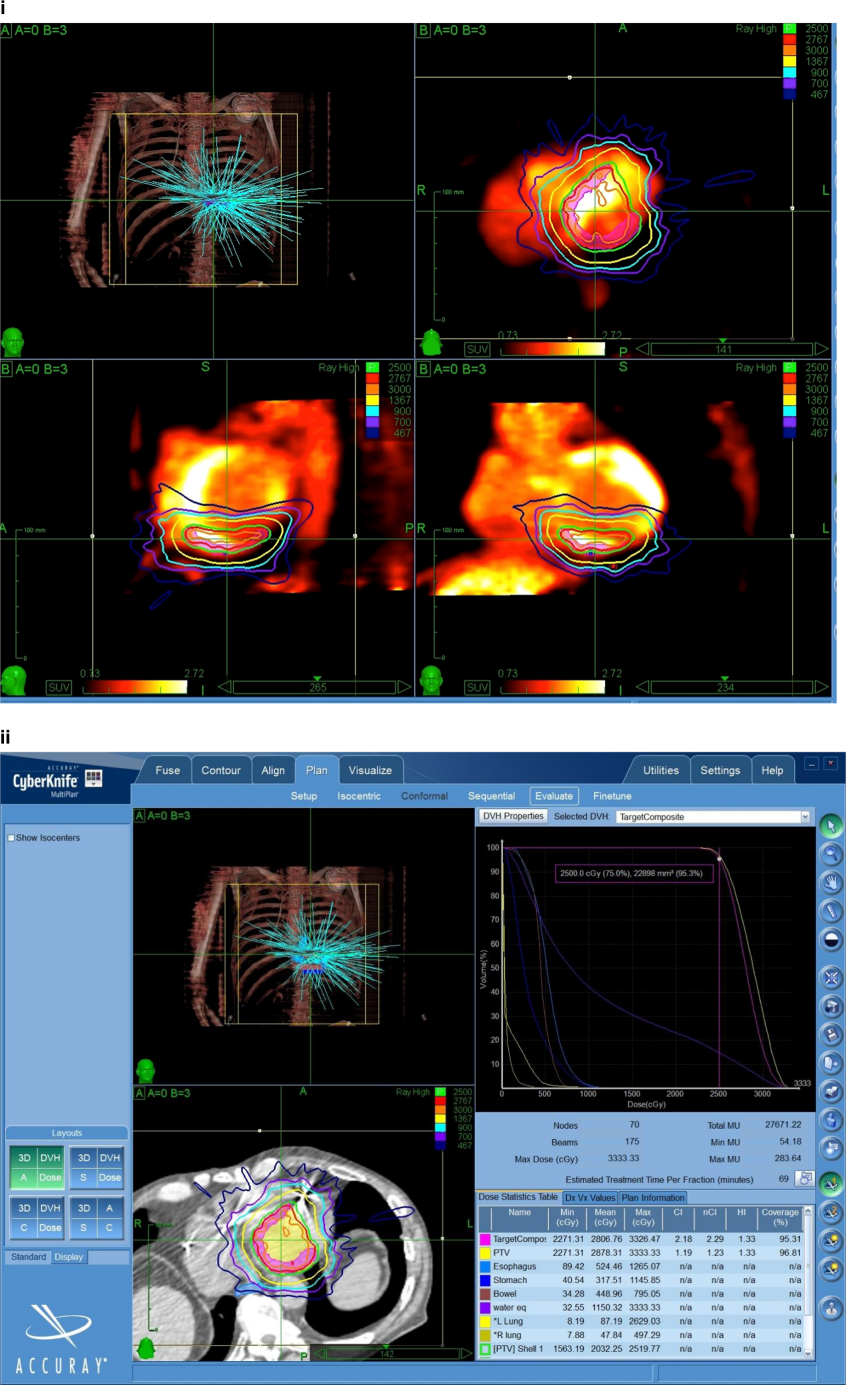
Treatment of a scar-based VT is shown. In part *i*, PET imaging demonstrating dense infarct in the inferobasal wall of the left ventricle (LV) is shown. The intended treatment plan is superimposed, along with planned delivery beams in three dimensions, as well as planned isodoses. In part (*ii*), the treatment software display is shown, with CT imaging of the intended inferobasal LV target shown. Superimposed on the CT image are delivered radiation beams and delivered isodoses

In Fig. 3, a representative example of a VT substrate target and treatment plan is shown. In this instance, an area of dense scar in the inferobasal left ventricle is demonstrated by PET scanning (Fig. 3(i)). Based on the inferobasal scar location, the designed treatment delivery to the arrhythmia substrate is superimposed, shown as isodose of calculated energy, with the central zone of treatment reaching 25 Gy and rapid dose falloff beyond. The resultant delivery plan is shown in Fig. 3(ii), with the planned delivery vectors and dosings of multiple low-energy radiation superimposed on a CT image of the same patient. A single dose maximum of 25 Gy was delivered in this example. Actual treatment time was 90 min.

Efficacy measured as reduction or elimination of VT has been demonstrated in all treated patients, ranging from no VT seen at follow-up to one patient with freedom from VT for several months before recurrence in the context of COPD exacerbation and possible pneumonia. No complications to date have been reported, with surveillance including follow-up echocardiography, chest radiographs, and clinical follow-up. However, a fixed dosing of 25 Gy has been used in all reported treatments; optimal dosing for specific patient populations remains undetermined. For AF patients, no complications were observed clinical and with serial chest imaging to date. Freedom from AF was observed, with one patient experiencing late AF recurrence (unpublished data).

## Future Directions

Additional clinical experience is clearly needed to further define safety and efficacy. At present, there are two active FDA-approved clinical studies evaluating STAR for the treatment of drug and catheter ablation refractory VT [21, 22]. In these pilot studies, patients with VT and structural heart disease and who have failed antiarrhythmic drug therapy and high-quality VT ablation are being enrolled for rescue therapy. The results of these studies will be eagerly anticipated. The clinical range of dosing energy relating to both efficacy and toxicity can likely benefit from further refinement. Applicability of treatment across all available delivery platforms is also yet to be defined. An additional technology that may prove useful as an adjunct to STAR is so-called body surface mapping, where intracardiac electrical activation can be mapped in reasonably accurate detail using an external array of skin-contact electrodes [13]. Potentially, this technology would allow for noninvasive mapping of arrhythmia substrate both pre- and post- treatment, which would help to further define and localize ablation targets, and to help better define efficacy. Further evaluation of this technology and its application to STAR is indicated.

In addition to treatment of cardiac arrhythmia substrates, stereotactic ablation can potentially be applied to therapy for other cardiac and non-cardiac targets. Septal ablation for treatment of LV outflow obstruction associated with hypertrophic cardiomyopathy, denervation of autonomic cardiac input for incessant VT, long QT syndromes, or catecholaminergic polymorphic ventricular tachycardia (CPVT) may be useful. Stellate ganglionic plexus ablation or renal autonomic denervation for hypertension may also be potential targets with this therapy.

## Conclusions

Stereotactic ablative radiotherapy is a well-established treatment paradigm that holds the promise to be a safe and effective therapy for cardiac arrhythmias in a completely noninvasive manner. Given the early stage of evaluation of this technology, more investigation and clinical experience are needed. Current preclinical and clinical experiences have suggested early efficacy and safety; however, additional clinical data under properly designed clinical trials are needed.
